# Association between weekend catch-up sleep and health-related quality of life of Korean adults

**DOI:** 10.1097/MD.0000000000014966

**Published:** 2019-03-15

**Authors:** Yun Hwan Oh, HyeonJu Kim, MiHee Kong, Bumjo Oh, Ji Hyun Moon

**Affiliations:** a Department of Family Medicine, Jeju National University Hospital, Jeju-si; b Department of Family Medicine, School of Medicine, Jeju National University, Jeju; c Department of Family Medicine, SMG-SNU Boramae Medical Center, Korea.

**Keywords:** chronotype, health-related quality of life, sleep debt, weekend catch-up sleep

## Abstract

Sleep debt is known to have harmful effects on health. Weekend catch-up sleep (CUS) is a behavior to cope with weekday sleep debt. However, it is unclear whether weekend CUS has advantageous effects on health because sleep hygiene guidelines recommend regularizing bed time and arousal time. The aim of this study was to identify whether weekend CUS behavior has an association with better health-related quality of life (HRQoL)

According to the inclusion criteria, 4871 participants were selected from the 2016 Korea National Health and Nutrition Examination Survey. Sleep questionnaires and European quality of life scale-5 dimensions (EQ-5D) questionnaire were used to collect data about the participants’ sleep patterns and HRQoL. Odds ratios (ORs) with 95% confidence intervals (95% CIs) for each dimensional problem of EQ-5D were derived by logistic regression. Mean EQ-5D index scores were compared between weekend CUS and non-CUS groups based on their weekday sleep durations and quintile of chronotype.

The ORs of dimensional problems of HRQoL of non-CUS group versus weekend CUS group were 1.63 (95% CI 1.07, 2.47) for usual activities, and 1.45 (95% CI 1.11, 1.90) for anxiety/depression. Mean EQ-5D scores of the weekend CUS group were significantly higher than those of the non-CUS group for sleeping less than 6 hours (0.953 ± 0.004 vs 0.936 ± 0.007, *P* = .036) and sleeping 6 to 7 hours (0.965 ± 0.003 vs 0.955 ± .0.004, *P* = .045). These findings were similar in the fourth quintile (Q4) of chronotype (0.965 ± 0.007 vs 0.951 ± 0.008, *P* = .008) and fifth quintile (Q5) (0.952 ± 0.006 vs 0.941 ± 0.007, *P* = .022).

Weekend CUS behavior was associated with better HRQoL than non-CUS among Korean adults. Especially, it was significant in participants who slept for less than 7 hours or participants whose chronotype was the fourth or fifth quintile. Attention may be needed for subjects with sleep short time and later chronotype who do not have weekend-CUS behaviors, because there is a risk that their HRQoL might be compromised.

## 1 Introduction

Sleep has an essential role in maintaining the proper functioning of the entire body system However, sleep problems are prevalent in the modern society,^[1]^ and sleep deficiency is one of the most common sleep problems.^[2]^ Sleep deficiency not only limits the proper functioning of daily life, but also increases the risk of metabolic diseases, such as hypertension,^[3]^ diabetes mellitus,^[4]^ obesity,^[5]^ and psychiatric problems like depression.^[6]^ A recent study reported that short sleep duration is associated with increased mortality and morbidity.^[7]^ Nowadays, chronic sleep deficiency and sleep disorder are considered public health problems.^[8]^

Weekend catch-up sleep (CUS) is one way to cope with insufficient sleep during workdays or weekdays by increasing the sleep duration during weekend or free days. However, weekend CUS is not solely explained by short sleep duration during workdays. An individual's chronotype may partly explain weekend CUS. Chronotype refers to an individual's preference for early or late sleep timing based on the circadian preference. Chronotype can be assessed by a few methods such as Morningness–Eveningness questionnaire and Munich Chrono-type questionnaire (MCTQ). Especially, the MCTQ is useful tool to identify individual's chronotype, because the MCTQ is consisted with separate questions about individual's sleep times on both weekday and weekend.^[9]^ Chronotype is calculated based on the midpoint between sleep onset and wake up. For this reason, the MCTQ provide quantitative measure of chronotype as a continuous variable. And the MCTQ is not based on subjective sleep preference but actual sleep behavior.^[9]^ It has been reported that evening chronotype persons have longer weekend sleep duration and weekend sleep extension despite having a similar weekday sleep duration as morning chronotype persons.^[10]^ For this reason, weekend CUS could be considered a compensatory sleep behavior for sleep debt caused by sleep duration and chronotype. Coping with sleep debt by weekend CUS might have beneficial effects on health by counteracting the harmful effects of sleep deficiency during weekdays. For example, previous studies have suggested that individuals with weekend CUS are at a lower risk for hypertension,^[11]^ obesity,^[12]^ and overall mortality.^[13]^

Health-related quality of life (HRQoL) can be a useful index to evaluate the effect of CUS on health. HRQoL not only indicates an individual's morbidities but also their subjective perception of their overall health in physical, mental, and social aspects. Sleep deficiency is associated with chronic diseases like stroke and cancer.^[14]^ Former studies have shown that sleep deficiency is associated with impairment in HRQoL.^[15]^ However, to our knowledge, no studies have investigated the association between weekend sleep extension and HRQoL.

This study aimed to examine the association between weekend CUS and HRQoL in the general adult population, then to reveal that weekend CUS have an association with better HRQoL.

## 2 Methods

### 2.1 Participants

Participants were selected based on raw data from the 2016 Korea National Health and Nutrition Examination Survey (KNHANES VII-1). The KNHANES is a nationwide representative cross-sectional survey conducted by the Korea Centers for Disease Control and Prevention. The KNHANES uses a stratified, multistage probability sampling design for the selection of household units. The selection was made from sampling units based on age, gender, and geographic area. The KNHANES consists of the health interview survey, health behavior survey, health examination survey, and nutrition survey. The health interview survey, health behavior survey, and nutrition survey are evaluated by self-administered questionnaires. Details of the study design and methods have been described elsewhere.^[16]^ Informed consent was obtained from all individual participants included in the study.

Of all the participants in KNHANES VII-1 (2016), 1768 individuals aged less than 19 years, and 590 participants who did not participate in the survey about sleep and HRQoL were excluded. In addition, 921 patients who had diseases that could strongly affect the sleep pattern and HRQoL, such as all types of cancer,^[17,18]^ coronary heart disease,^[18,19]^ stroke,^[18,20]^ liver cirrhosis,^[21,22]^ chronic kidney disease, depression, arthritis (osteoarthritis and rheumatoid arthritis) were excluded. Finally, a total of 4871 participants (2160 men and 2711 women) were included in the analyses.

### 2.2 Measurements

#### 2.2.1 Sleep duration, chronotype, social jetlag, and weekend CUS

Participants’ average weekday and weekend sleep durations were calculated based on their responses to the following questions: “On a weekday (or working day), at what time do you go to sleep and at what time do you get up? On a weekend (or the day when you do not work, the day before you do not work), at what time do you go to sleep and at what time do you get up?” Average sleep duration was calculated by the following formula: (weekday sleep duration × 5 + weekend sleep duration × 2)/7.

To quantify chronotype and social jetlag, we extracted and used several variables, such as midpoint of sleep on free days (MSF), midpoint of sleep on workdays (MSW), sleep duration on workdays (SDW), and sleep duration on free days (SDF) using the sleep-related questions mentioned above. “Midpoint of sleep on free days corrected for sleep extension on free days (MSFsc)” is used as an indicator of chronotype.^[23]^ Each participant's MSFsc was calculated using the equation given below.^[24]^ MSFsc is represented as local time. If sleep duration on free days was shorter than or equal to sleep duration on work days, MSFsc is equal to MSF. The categorization of chronotype was based on quintiles of the MSFsc, wherein early chronotype was the first quintile (lowest MSFsc, Q1), intermediate chronotype was the second (Q2), third (Q3), and fourth (Q4) quintiles, and the late chronotype was the fifth quintile (highest MSFsc, Q5).^[25]^ 
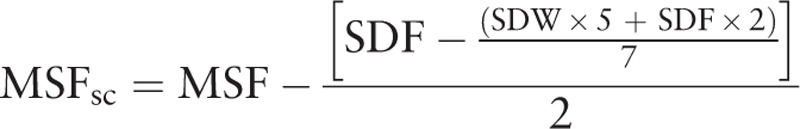


Social jetlag is the difference between the MSF–MSW).

Weekend CUS duration was calculated as the weekend sleep duration minus the weekday sleep duration. Weekend CUS was defined as sleep duration in the weekend being longer than that in weekdays. Weekday sleep duration was categorized into 5 groups (sleep duration <6 hours, 6 to <7 hours, 7 to <8 hours, 8 to <9 hours, ≥9 hours).

#### 2.2.2 HRQoL and perceived health status

The KNHANES uses the Korean version of European quality of life scale-5 dimensions (EQ-5D) questionnaire^[26]^ to assess HRQoL. The EQ-5D consists of 5 dimensions of the current health status. These dimensions are mobility, self-care, usual activities, pain/discomfort, and anxiety/depression. Each dimensional status in terms of severity is rated as “no problems,” “some problems,” or “extreme problems.” This study used the Korean version of weighted model of the EQ-5D for the analyses. Reliability and validity of the Korean version of the EQ-5D were evaluated and verified by a previous study on general population and the reliability level was moderate (Cohen kappa 0.32–0.64).^[27]^ Apart from the questionnaire about 5 dimensions, the EQ-5D index score is a single index value that indicates health status. In this study, we used the EQ-5D index score, which was calculated and validated by the previous studies^[28,29]^ using their estimated weighted quality value for Koreans.

Perceived health status was inferred based on the following question, “What is your perception of your overall health?” (“very good,” “good,” “fair,” “poor,” “very poor”). The responses “very good,” “good,” and “fair” were considered to indicate “not poor,” and “poor” and “very poor” were considered to indicate “poor.”

#### 2.2.3 Anthropometric, demographic, and lifestyle factors

Participants’ height (cm) and body weight (kg) were measured, and their body mass index (BMI) was calculated as weight (in kilograms) divided by the square of the height (in meters).

The demographic variables (age, sex, education level, marital status, and monthly income) and lifestyle factors (smoking status, alcohol consumption, and physical activity level) of the participants were collected through a survey. Education level was categorized as middle school or lower, high school, and college or higher. Marital status was categorized as single, married, and separated/divorced/widowed. Monthly income was divided into quartiles, and categorized into low, low-middle, high-middle, and high income categories. For smoking status, participants were classified as current smoker, exsmoker, or never smoked. High-risk drinkers were defined as men consuming >14 glasses of alcohol per week, and women consuming >7 glasses of alcohol per week.

#### 2.2.4 Physical activity

Physical activity level was evaluated by the Korean global physical activity questionnaire (GPAQ).^[30]^ Each participant's physical activity level was quantified into metabolic equivalent of task-minute per week and was classified into 3 levels (high, moderate, or low) according to the analysis and calculation guidelines of the GPAQ.^[31]^

#### 2.2.5 Charlson comorbidity index score

For each participant, to evaluate the overall health status, a comorbidity score was calculated by the Charlson comorbidity index (CCI).^[32]^ The comorbidities used to calculate the CCI were identified and obtained through the survey. The CCI score was calculated for each participant as the total of the participant's comorbid conditions which have been weighted. Comorbid conditions with 1 point are myocardial infarction, congestive heart failure, peripheral vascular disease, cerebrovascular disease, dementia, chronic obstructive pulmonary disease, connective tissue disease, gastrointestinal ulcer disease, mild liver disease and diabetes mellitus. Conditions with 2 points are moderate to severe renal disease, diabetes with end-organ damage, hemiplegia, and any kind of malignancy. Moderate to severe liver disease was given 3 points. Metastatic solid tumor or AIDS was given 6 points. The point values were summed for a total number, to which 1 point was added for each decade greater than forty years of age.

### 2.3 Statistical analysis

All participants were divided into 2 groups (weekend CUS group and non-CUS group). To compare the basic demographics, anthropometric characteristics, lifestyle factors, and the 5 dimensions of quality of life using the EQ-5D of the 2 groups, continuous variables were presented as estimated mean ± standard errors and compared using *T* test, while the categorical variables were presented as unweighted numbers and estimated percentages and compared using the chi-squared (*χ*^2^) test.

Second, univariate and multivariate logistic regression analyses were performed to evaluate the association between weekend CUS and each dimensional status of quality of life. We modified the original 3 categories into 2 categories by combining the categories “some problems” and “extreme problems” as the number of “extreme problems” responses for each dimension was very less to perform the analyses.

Third, to evaluate the influence of weekday sleep duration and chronotypes, unadjusted and adjusted mean EQ-5D index scores of the 2 groups were compared according to the weekday sleep duration categories and chronotype quintiles.

For data management and analysis, STATA version 13.0 (StataCorp, College Station, TX) was used. The *P*-values of all the results reported below are bilateral, and the significance level was set at *P* < .05.

## 3 Results

### 3.1 Baseline characteristics of the study participants

Table 1 shows the baseline characteristics of the study participants based on the presence or absence of weekend CUS. Of a total of 4871 participants, the weekend CUS group comprised of 2164 subjects (44.2%), and non-CUS group comprised of 2707 subjects (55.8%). According to comparison of the 2 groups, significant differences were seen for most variables between groups.

**Table 1:**
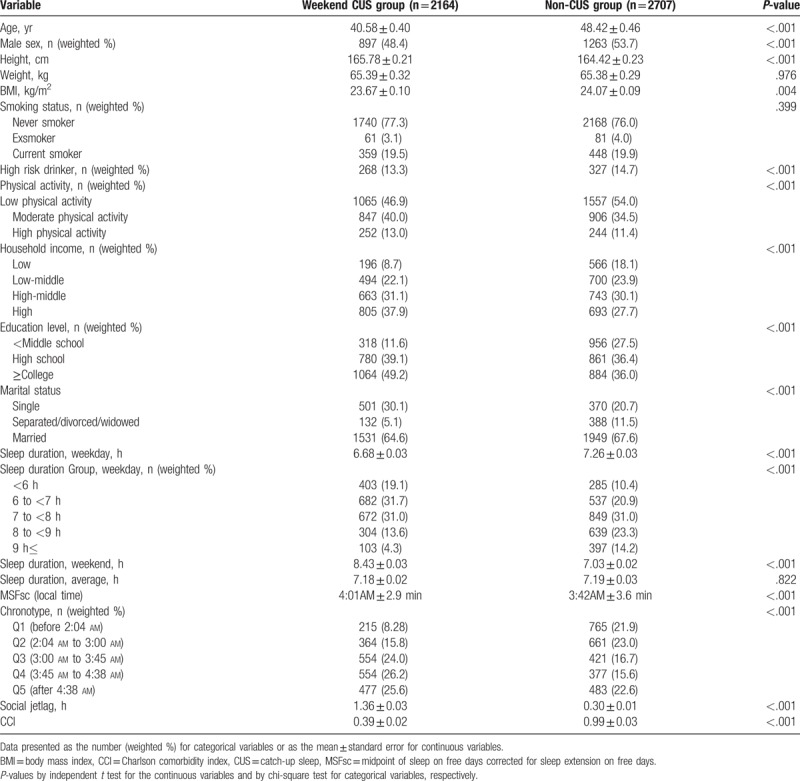
Baseline characteristics of study participants according to weekend catch-up sleep.

Participants of the weekend CUS group were significantly younger than those of the non-CUS group (40.58 ± 0.40 years vs 48.42 ± 0.46 years, *P* < .001). The proportion of men in weekend CUS group was lesser than that of the non-CUS group (48.4% vs 53.7%). Further, the BMI was lower in the weekend CUS group than in the non-CUS group (23.67 ± 0.10 kg/m^2^ vs 24.07 ± 0.09 kg/m^2^, *P* = .001). The weekend CUS group had shorter sleep duration than did the non-CUS group on weekdays (6.68 ± 0.03 hours vs 7.26 ± 0.03 hours, *P* < .001). On the other hand, weekend sleep duration of the weekend CUS group was longer than that of the non-CUS group (8.43 ± 0.03 hours vs 7.03 ± 0.02 hours, *P* < .001). However, there was no significant difference in the average sleep duration between the groups (7.18 ± 0.02 hours vs 7.19 ± 0.03 hours, *P* = .822). The weekend CUS group were later chronotype compared to the non-CUS group (4:01 am ± 2.9 minutes vs 3:42 am ± 3.6 minutes, *P* < .001). Moreover, social jetlag was longer in the weekend CUS group than in the non-CUS group (1.36 ± 0.03 hours vs 0.03 ± 0.01 hours, *P* < .001). The weekend CUS group had lower CCI than that of the non-CUS group (0.39 ± 0.02 vs 0.99 ± 0.03, *P* < .001).

### 3.2 Comparison of the HRQoL, perceived health status, and PHQ-9 (depression) according to weekend CUS

Table 2 shows the distribution of the HRQoL, perceived health status, and the PHQ-9 scores based on the presence or absence of weekend CUS. The proportions of “Have problems” response in every EQ-5D dimension were higher in the non-CUS group than in the weekend CUS group. The EQ-5D index score was lower in the non-CUS group than in the CUS group (0.975 ± 0.001 vs 0.958 ± 0.001, *P* < .001). Further, a higher number of participants in the non-CUS group rated their own health status as poor than did participants in the weekend CUS group (12.1% vs 16.7%, *P* < .001). The PHQ-9 score was higher in the non-CUS group but not statistically significant (2.41 ± 0.08 vs 2.59 ± 0.09, *P* = .087).

**Table 2:**
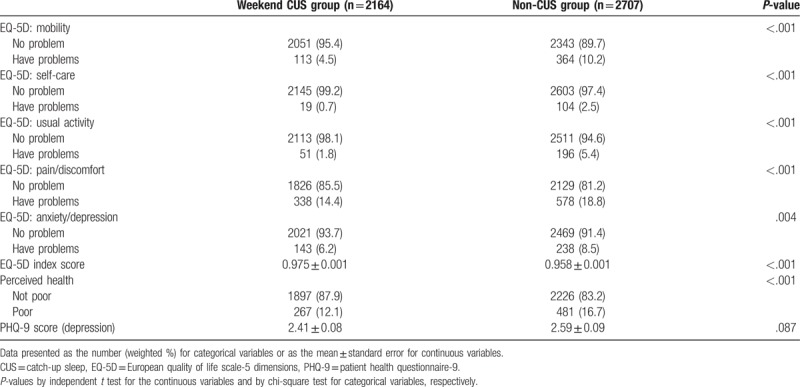
Comparison of health-related quality of life, perceived health, and depression according to weekend catch up sleep.

### 3.3 Association between weekend CUS and HRQoL

Table 3 presents the odds ratios (ORs) and 95% confidence intervals (CIs) for poor HRQoL and poor perceived health status on all 5 EQ-5D dimensions (mobility, self-care, usual activities, pain/discomfort, anxiety/depression) based on the presence of weekend CUS as the independent variable. In the crude logistic analyses, the ORs for problematic HRQoL increased for the non-CUS group on all 5 dimensions of the EQ-5D. Moreover, the ORs for poor perceived health status also increased for the non-CUS group. Following the crude logistic regression analyses, we performed multivariate logistic regression analyses, adjusting for CCI, sex, BMI, physical activity, alcohol consumption, smoking status, household income, education level, and marital status. The OR for having problems in the dimensions of usual activities (OR = 1.63, 95% CI 1.07, 2.47), and anxiety/depression (OR = 1.45, 95% CI 1.11, 1.90) significantly increased for the non-CUS group. Further, the OR for poor perceived health status (OR = 1.25, 95% CI 1.02, 1.54) increased for the non-CUS group.

**Table 3:**
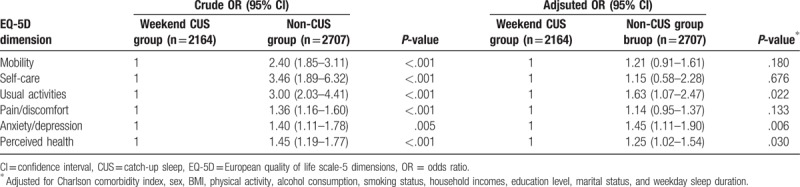
ORs and 95% CI for impaired health-related quality of life and self-reported health status according to weekend catch up sleep.

### 3.4 Association between weekday sleep duration and HRQoL

**Figure 1:**
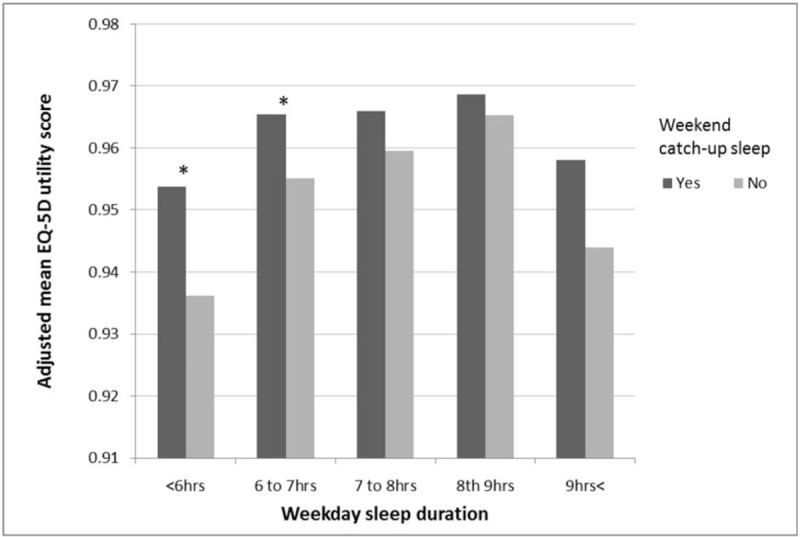
Means of EQ-5D index score, according to weekday sleep duration and weekend CUS. The bars from left to right are week day sleep duration <6 h, 6 to 7 h, 7 to 8 h, 8 to 9 h, 9 h < in weekend CUS group and non-CUS group. Means are adjusted for Charlson comorbidity index, sex, BMI, physical activity, alcohol consumption, smoking status, household incomes, education level, and marital status. ^∗^*P*-value < .05. BMI = body mass index, CUS = catch-up sleep, EQ-5D = European quality of life scale-5 dimensions.

To evaluate the effect of weekday sleep duration on the HRQoL, we compared the adjusted mean EQ-5D index scores of the 2 groups according to the weekday sleep hours. Figure 1 shows the mean EQ-5D index score of each group by weekday sleep duration categories. After adjusting for CCI, sex, BMI, physical activity, alcohol consumption, smoking status, household incomes, education level, and marital status, the mean adjusted EQ-5D score of the weekend CUS group was higher in participants who slept for less than 6 hours (0.953 ± 0.004 vs 0.936 ± 0.007, *P* = .036) and participants who slept 6 to 7 hours (0.965 ± 0.003 vs 0.955 ± .0.004, *P* = .045).

### 3.5 Association between chronotype and HRQoL

**Figure 2:**
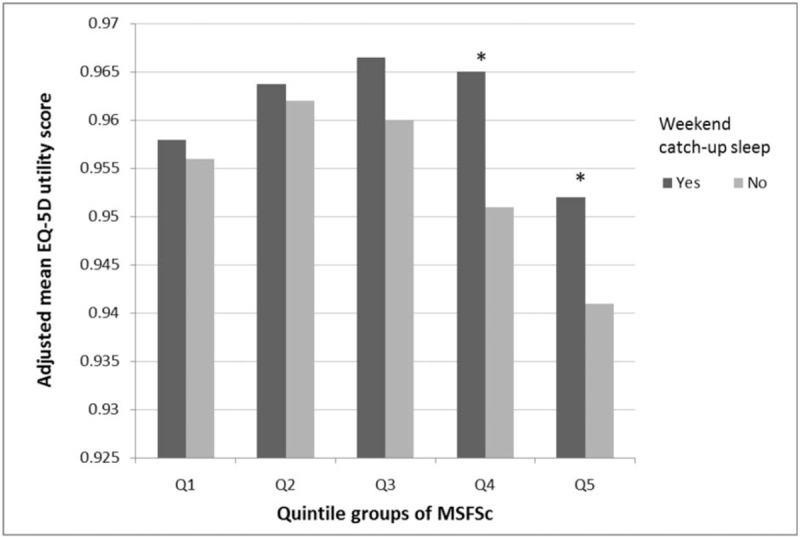
Means of EQ-5D index score, according to chronotype and weekend CUS. The bars from left to right are quintile of MSFsc: Q1 (lowest), Q2, Q3, Q4, and Q5 (highest). Means are adjusted for Charlson comorbidity index, sex, BMI, physical activity, alcohol consumption, smoking status, household incomes, education level, marital status, and weekday sleep duration. ^∗^*P*-value < .05. BMI = body mass index, CUS = catch-up sleep, EQ-5D = European quality of life scale-5 dimensions, MSFsc = midpoint of sleep on free days corrected for sleep extension on free days.

We also compared the adjusted mean EQ-5D index scores of the 2 groups according to the chronotype, categorized by quintile of MSFsc (Fig. 2). After adjusting for CCI, sex, BMI, physical activity, alcohol consumption, smoking status, household incomes, education level, marital status, and weekday sleep duration, the mean adjusted EQ-5D scores of the weekend CUS group were higher in Q4 (0.965 ± 0.007 vs 0.951 ± 0.008, *P* = .008) and Q5 (0.952 ± 0.006 vs 0.941 ± 0.007, *P* = .022) participants.

## 4 Discussion

The purpose of the study was to investigate whether weekend CUS behavior is associated with higher HRQoL than non-CUS behavior in Korean adults. To our knowledge, this study might be the first to examine the association between weekend CUS behavior and HRQoL. Further, we investigated the additional factors influencing weekend sleep extension, such as weekend sleep duration and chronotypes. The results of our study suggested some interesting associations among HRQoL, weekend CUS behavior, weekday sleep duration, and chronotype.

First, according to our logistic analyses, the univariate analysis showed significant higher ORs on every EQ-5D dimension with problem and poor perceived health status. However, the multivariate analysis showed significant higher ORs only on 2 dimensions (usual activities, and anxiety/depression) of the EQ-5D and perceived health status. There are 2 possible explanations for these results. The first is that the sleep debt of the CUS group accumulated during weekday does not deteriorate their physical health status significantly compared to the non-CUS group. The second explanation is that CUS behavior is not enough to compensate for the debased physical health status as a result of sleep debt. It is difficult to identify the exact reason for this based on the findings of this study. However, the average sleep duration during weekday was shorter in the weekend CUS group than that in the non-CUS group, and therefore, sleep debt might have been more for the weekend CUS group. The ORs were higher for the non-CUS group although not statistically significant. In sum, there might be some positive association between CUS behavior and HRQoL, but not enough in terms of physical health status.

Sleep debt has been known to lead to several health conditions and consequences.^[33]^ However, these effects tend to be seen in the psychological aspects, such as mood, compared to cognitive or motor performances.^[34]^ The 5 dimensions of the EQ-5D represent the physical, mental health, and social functioning.^[35]^ Mobility and self-care dimensions represent physical health status. Pain/discomfort and anxiety/depression dimension reflect mental health status. Usual activities, such as work, study, housework, and family or leisure activities mainly reflect social functioning. Since we excluded participants with severe diseases which could influence the HRQoL and sleep pattern, the possible underlying physical disabilities of the study participants were not prominent. Therefore, weekday sleep debt alone might not be a critical factor disrupting mobility and self-care. However, its impact on mental health and social functioning are substantial. Our findings are mostly consistent with those of previous researches.^[34]^

Second, participants with a short weekday sleep duration (less than 7 hours) showed significant differences in the HRQoL between weekend CUS group and non-CUS group. This might be because participants with short weekday sleep duration have more sleep debt than do others. This finding represents what we expected at the beginning of the study—weekend CUS pattern might associate with better HRQoL. It can be explained by compensating role of weekend CUS for sleep debt caused by lack of weekday sleep duration, but it is hard to confirm the causal relationship between weekend CUS and better HRQoL, because of limitation of the study design.

Finally, we found that the HRQoL of specific chronotypes (Q4, Q5) were more significantly associated with weekend CUS than those of other chronotypes. This finding was obtained after adjusting for several factors affecting the HRQoL and weekday sleep duration. Therefore, discrepancies in the HRQoL between weekend CUS group and non-CUS group for Q4, Q5 chronotypes might be not because of sleep debt due to the lack of weekday sleep duration. According to a previous study,^[10]^ evening types accumulate more sleep debt during weekdays although their sleep needs and durations are similar to the morning types. For this reason, we expected that later chronotypes accumulate more sleep debt and the compensatory effect of weekend CUS on the HRQoL might be larger in later chronotypes. Our study findings that later chronotypes with weekend CUS are associated with better HRQoL than non-CUS and that there was no definite association between CUS behavior and HRQoL in relatively earlier chronotypes are mostly consistent with previous study findings.

It is known that different types of sleep deprivation have several short-term and long-term health effects that alter the metabolic, immunologic, hematologic, nervous, and hormonal systems, as well as deteriorate mental and physical health.^[36]^ Therefore, insufficient sleep is also associated with impairment of the HRQoL.^[15]^ However, it is not clear whether weekend sleep extension behavior associates with better HRQoL because the well-known clinical principles—the sleep hygiene rules^[37]^ recommend regularizing the bedtime and arousal time for better sleep quality and quantity. Weekend CUS behavior could be considered a violation of the sleep hygiene rules. Our study found that weekend CUS is associated with better HRQoL especially for participants who sleep for less than 7 hours on weekdays or later chronotypes.

This study has a few limitations, and therefore, the findings of this study should be interpreted with caution. First, the cross-sectional design of the study makes it difficult to identify the causal relationship between weekend CUS behavior and HRQoL. It is difficult to confirm whether weekend CUS behavior directly promotes HRQoL. Second, in this dataset, there were no data about the subjective sleep quality or excessive daytime sleepiness. If those data were included, they could be used as an indicator of subjective sleep debt, whereby the compensatory roles of CUS behavior might become clearer. Furthermore, there were no available data about sleep disorders, such as obstructive sleep apnea or insomnia, which the some proportions of study participants had. Therefore, there is a limitation to assessing the sleep quality, which is irrelevant to sleep duration or chronotypes. Despite these limitations, our study is unique because the results of our study were representative of the general population of the entire nation.

There is a need for further research that takes into account subjective sleep quality and presence of several sleep disorders. Furthermore, a longitudinal study might identify a causal relationship between CUS behavior and HRQoL.

## 5 Conclusion

Weekend CUS behavior was associated with better HRQoL compared to non-CUS. Especially, participants with short weekday sleep duration and late chronotypes showed significantly better HRQoL. Clinicians should pay attention to people with short sleep duration and late chronotypes who don’t have weekend CUS behavior, for assessing and improving the HRQoL of those people.

## Author contributions

Yun Hwan Oh wrote the manuscript and performed the statistical analyses, Mi Hee Kong and Hyeon Ju Kim contributed to the conception and design of this study protocol, Bumjo Oh provided methodological support, and Ji Hyun Moon was responsible for the study design and helped interpret the data. All authors are responsible for the entire contents of this article and have approved the submission of the manuscript.

**Conceptualization:** Hyeon Ju Kim, MiHee Kong.

**Data curation:** Hyeon Ju Kim, MiHee Kong.

**Formal analysis:** Yun Hwan Oh.

**Funding acquisition:** Yun Hwan Oh.

**Investigation:** Yun Hwan Oh.

**Methodology:** Bumjo Oh.

**Project administration:** Yun Hwan Oh, Ji Hyun Moon.

**Resources:** Yun Hwan Oh.

**Software:** Ji Hyun Moon.

**Supervision:** Ji Hyun Moon.

**Validation:** Yun Hwan Oh.

**Visualization:** Yun Hwan Oh.

**Writing – original draft:** Yun Hwan Oh.

**Writing – review and editing:** Yun Hwan Oh.
